# Supplementary material for: Self-reported access to specialty clinics and receipt of health surveillance among U.S. patients with neurofibromatosis 1: a national survey
Source: Orphanet J Rare Dis. 2025 Apr 16;20:185. doi: 10.1186/s13023-025-03677-5 (PMC12004880; doi:10.1186/s13023-025-03677-5)
Supplement: Supplementary file 1 — Supplementary material 1. [file 13023_2025_3677_MOESM1_ESM.docx]

**Appendix. Online-Only Supplemental Tables**

Supplemental Table 1. Characteristics of Survey Respondents and Eligible NF Registry Enrollees

|  | Survey Respondents^a^  (n=322) | Eligible NF Registry Enrollees^a^ (n=6908) | p-value |
| --- | --- | --- | --- |
| Age: n (%), mean  Children (age 0-17 years)  Adults (age 18+ years) | 162 (50.3%), 7.5 years  160 (49.7%), 43.8 years | 2776 (40.2%), 10.4 years  4132 (59.8%),  40.2 years | p<0.001  p<0.001  p<0.001  p=0.003 |
| Gender, n (%)  Female  Male | 187 (58.1%)  135 (41.9%) | 3902 (56.5%)  3006 (43.5%) | p=0.55 |
| Race/Ethnicity^b^  White  Native American or Alaskan Native  Black, Afro-Caribbean, or African-American  Native Hawaiian or other Pacific Islander  Latino/Hispanic  East Asian  South Asian  Other | 252 (78.3%)  7 (2.2%)  18 (5.6%)  0 (0.0%)  13 (4.0%)  11 (3.4%)  5 (1.6%)  16 (5.0%) | 5277 (76.6%)  198 (2.9%)  531 (7.7%)  42 (0.6%)  247 (3.6%)  189 (2.7%)  103 (1.5%)  304 (4.4%) | p=0.61 |
| US Region^c^  Far West  Rocky Mountain  Southwest  Plains  Great Lakes  Southeast  Mid-Atlantic  New England | 60 (18.8%)  22 (6.9%)  31 (9.7%)  20 (6.3%)  40 (12.5%)  53 (16.6%)  69 (21.6%)  24 (7.5%) | 1020 (15.0%)  434 (6.4%)  676 (9.9%)  523 (7.7%)  1092 (16.1%)  1645 (24.2%)  1041 (15.3%)  360 (5.3%) | p=0.001 |
| Attendance at NFCN clinic  Yes  No  Missing | 199 (61.8%)  85 (26.4%)  38 (11.8%) | 2343 (33.9%)  2545 (36.8%)  2020 (29.2%) | p<0.001 |

Note: P-values calculated using t-test for gender and age, and chi-square for ancestry, clinic type, and region. ^a^ All reported characteristics are of individuals with NF1 (adult survey respondents and children of parent/caregiver survey respondents). ^b^ Does not include 17 individuals with missing ancestry. ^c^ Does not include 108 individuals with missing US Region.

Supplemental Table 2. Self-reported Reasons for Not Seeking Care at a Specialized NF Clinic

|  | Adults with NF1 | | | | Parents of Children with NF1 | | | |
| --- | --- | --- | --- | --- | --- | --- | --- | --- |
|  | Major Problem | Minor Problem | Not a Problem | Missing | Major Problem | Minor Problem | Not A Problem | Missing |
| Insurance doesn’t cover specialized clinics or they are out of network | 19% | 21% | 45% | 15% | 7% | 27% | 57% | 9% |
| Didn’t know there were specialized NF clinics | 18% | 22% | 45% | 15% | 17% | 17% | 58% | 9% |
| Don’t want to travel to specialized NF Clinic/ too far away | 15% | 24% | 46% | 15% | 8% | 56% | 28% | 7% |
| Can’t travel to specialized NF clinic (due to my health, cost of travel, etc) | 15% | 14% | 56% | 15% | 11% | 15% | 66% | 9% |
| Don’t see the value in attending a specialized NF clinic at this time | 6% | 17% | 62% | 15% | 4% | 35% | 52% | 9% |

Note: All percentages are weighted using inverse propensity scores based on age, region, and NFCN clinic attendance within responses from 88 adults and 45 parents of children with NF1 who did not attend or were unsure whether they attended a specialized NF clinic in the prior 3 years.
